# Whole-genome characterization and molecular epidemiology of Feline coronavirus (FeCoV) circulating in domestic cats in Thailand: First report of FeCoV-II whole genomes

**DOI:** 10.14202/vetworld.2025.3888-3901

**Published:** 2025-12-14

**Authors:** Yu Nandi Thaw, Kamonpan Charoenkul, Chanakarn Nasamran, Ekkapat Chamsai, Waleemas Jairak, Eaint Min Phyu, Hnin Wai Phyu, Supassama Chaiyawong, Somsak Pakpinyo, Alongkorn Amonsin

**Affiliations:** 1 Department of Veterinary Public Health, Faculty of Veterinary Science, Chulalongkorn University, Bangkok, Thailand; 2 Center of Excellence for Emerging and Re-emerging Infectious Diseases in Animals, Faculty of Veterinary Science, Chulalongkorn University, Bangkok, Thailand

**Keywords:** domestic cats, FeCov, genotype I and II, molecular epidemiology, phylogenetic analysis, *spike* (*S*) gene, Thailand, whole-genome sequencing

## Abstract

**Background and Aim:**

Feline coronavirus (FeCoV) is a widely circulating Alphacoronavirus that causes mild enteric infections and, in some cases, progresses to Feline infectious peritonitis, a fatal systemic disease. FeCoV consists of two genotypes (I and II) and two biotypes (FeCoV and **feline infectious peritonitis virus** [FIPV]). Despite its importance, whole-genome data, particularly for FeCoV genotype II, remain limited in Thailand. This study aimed to determine the prevalence of FeCoV in domestic cats and to genetically characterize circulating strains using whole-genome and *S* gene sequencing.

**Materials and Methods:**

A total of 471 rectal swabs were collected from domestic cats presented to private small animal hospitals in Bangkok and neighboring provinces from October 2022 to October 2023. FeCoV detection and genotyping were performed using one-step reverse transcription polymerase chain reaction targeting the 3′UTR and *S* gene, respectively. Selected FeCoV-positive samples were subjected to whole-genome sequencing (WGS) (n = 4) and complete S gene sequencing (n = 6) using Oxford Nanopore technology with Minimap2, Racon, and Medaka pipelines. Phylogenetic and genetic analyses were conducted using MEGA program.

**Results:**

FeCoV positivity was 21.87% (103/471), with higher detection in young cats (<6 months; 28.46%), though age, clinical status, and season showed no significant association (p > 0.05). Genotype I was overwhelmingly predominant (99.03%), whereas genotype II was rare (0.97%). Phylogenetic analysis revealed that Thai FeCoV-I strains clustered closely with Chinese and Dutch FeCoV-I strains, while the FeCoV-II strain grouped with Chinese FeCoV-II. Whole-genome pairwise comparisons showed high nucleotide and amino acid identities with their respective genotype references. No mutations were detected in the S1/S2 or S2 cleavage sites of Thai FeCoV-I, indicating conserved spike characteristics typical of FECoV biotypes. FeCoV-II exhibited the characteristic deletion and insertion patterns known for this genotype. No evidence of recombination with other coronaviruses was observed.

**Conclusion:**

This study provides updated molecular epidemiology of FeCoV in Thailand and reports the **first complete FeCoV-II genome sequences** from the country. The predominance of FeCoV-I and the detection of conserved spike regions highlight the need for genotype-specific surveillance and the reconsideration of vaccine strategies that currently target FeCoV-II. Expanded nationwide monitoring and detailed recombination analyses are warranted to better understand FeCoV evolution and transmission in feline populations.

## INTRODUCTION

Coronaviruses (CoVs) are enveloped, nonsegmented, single-stranded RNA viruses belonging to the family Coronaviridae. Their genomes contain 11 open reading frames (ORFs) arranged in the order 5′UTR–ORF1a–ORF1b–S–ORF3a/b/c–E–M–N–ORF7a/b–3′UTR [1, 2]. CoVs are classified into four genera: Alphacoronavirus, Betacoronavirus, Gammacoronavirus, and Deltacoronavirus. Within the Alphacoronavirus genus, the Alphacoronavirus 1 species comprises canine enteric coronavirus (CECoV) and feline coronavirus (FeCoV), both of which primarily infect dogs and cats, respectively, and are commonly associated with gastroenteritis and diarrhea in affected animals [3].

FeCoV comprises two major genotypes, type I (FeCoV-I) and type II (FeCoV-II), which are differentiated by genetic divergence in the spike (*S*) gene [4, 5]. FeCoV is also divided into two biotypes: the feline enteric coronavirus (FeCoV) and the highly pathogenic Feline infectious peritonitis (FIP) virus (FIPV) [6]. FeCoV is widespread and typically causes mild to moderate gastroenteritis, whereas FIPV results in FIP, a fatal systemic disease of felids. FIP occurs in two clinical forms: the wet (effusive) form, characterized by peritonitis and/or pleuritis, and the dry (non-effusive) form, which features granulomatous lesions affecting organs such as the central nervous system and eyes. FIPV is believed to emerge from mutations in FeCoV within infected hosts; however, the precise genetic determinants driving this biotype transition remain incompletely understood [7, 8].

Previous studies conducted in Thailand have primarily focused on FeCoV genotype I (FeCoV-I), examining partial and complete genomic regions, yet no whole-genome sequencing (WGS) of FeCoV-II has been performed to date [9–12]. This represents a critical gap because FeCoV-II, although less frequently detected, plays an important role in viral evolution and may arise through recombination between FeCoV-I and canine coronaviruses. A similar pattern is observed across other Asian countries. Reports from China [13, 14], Indonesia [15–17], Malaysia [18], and Vietnam [19] have also emphasized FeCoV-I, with FeCoV-II characterized primarily through partial *S* gene sequencing rather than complete genomic analysis. As a result, the full genomic diversity, recombination potential, and evolutionary relationships of FeCoV-II remain poorly understood in the region. This lack of whole-genome data restricts our ability to track genotype shifts, detect recombination events, and identify genomic determinants associated with pathogenicity, including those distinguishing FeCoV from FIPV. Given the scarcity of comprehensive molecular data on circulating FeCoVs in Thailand, particularly FeCoV-II, there is a significant gap in understanding the epidemiology, molecular evolution, and genetic characteristics of FeCoVs in Thai domestic cats.

To address this gap, the present study aimed to conduct a cross-sectional survey and whole-genome characterization of FeCoVs circulating in domestic cats in Thailand. Building on the limitations identified in previous studies [9–19], this research sought to determine FeCoV occurrence using reverse transcription polymerase chain reaction (PCR) (RT-PCR), classify circulating strains into FeCoV-I and FeCoV-II genotypes through *S* gene–based genotyping, and generate WGS and complete *S* gene sequences for selected FeCoV-positive samples. By applying WGS, the study aimed to provide a more complete understanding of the genomic features, phylogenetic relationships, and molecular diversity of Thai FeCoVs. The resulting genomic data are intended to support improved surveillance, facilitate early detection of genotype shifts or recombination events, and contribute to the development of more effective prevention, diagnostic, and vaccination strategies for FeCoV infection in Thailand.

## MATERIALS AND METHODS

### Ethical approval

This study was conducted strictly in accordance with the ethical principles, guidelines, and institutional regulations governing the use of animals in research at Chulalongkorn University. All procedures involving animals were reviewed and approved by the Chulalongkorn University Animal Care and Use Committee (CU-ACUC) under protocol numbers CU-VET IACUC#2031025 and 2331076. The protocol covered sample collection, handling, transportation, and laboratory processing of biological materials.

As the sampling involved non-invasive rectal swabs collected from client-owned domestic cats during routine veterinary visits, no experimental manipulation, sedation, or procedures causing pain, distress, or behavioral alteration were performed. Sample collection was carried out exclusively by licensed veterinarians or trained veterinary personnel to ensure animal welfare and procedural compliance. The study adhered to all institutional and national standards for minimizing animal discomfort and ensuring responsible use of animals in research.

Prior to sample collection, verbal informed consent was obtained from all pet owners or guardians after providing clear information regarding the study purpose, sampling procedures, potential benefits, and assurance of confidentiality. Participation was entirely voluntary, and owners retained the right to withdraw their animals at any stage without affecting veterinary services. No incentives were offered for participation.

This research fully followed the Animal Research: Reporting of *In Vivo* Experiments 2.0 guidelines for reporting animal research and complied with the ethical frameworks outlined in the Guide for the Care and Use of Laboratory Animals, the Thai Animal Welfare Act, and the institutional policies of the Faculty of Veterinary Science, Chulalongkorn University. All samples were anonymized and coded to protect the identity of both owners and their animals.

### Study period and location

A cross-sectional study was conducted from October 2022 to October 2023 at private small animal hospitals in Bangkok, Nonthaburi, and Samut Prakan.

### Calculation of sample size and sample collection

The sample size was calculated as described by Cochran [20]. The sample size calculation was based on the parameters, including the prevalence (p-value) of CoV in cats (at 31%) [10]. The confidence interval (z value) was 95%, and the precision (d value) was 0.045. Thus, the minimum required sample size was 406. A total of 471 samples were collected to increase the reliability of the sample size, which exceeded the required number. In this study, 471 rectal swab samples were collected from cats (n = 471) using convenience sampling. The samples were collected regardless of the age, sex, breed, or clinical status of the cats. Demographic data, including age, sex, breed, and clinical status, were collected and recorded from both hospital patient records and pet owners while collecting the samples. The collected swabs were placed in viral transport media (Eagle’s Minimum Essential Medium) and temporarily stored in a refrigerator at 4°C at the animal hospital, then transported to the laboratory within 24 h.

### RNA extraction

RNA was extracted from the rectal swab sample using the GeneAll® GENTiTM Viral DNA/RNA Extraction Kit (GeneAll®; Lisbon, Portugal) on a GENTiTM 32 (GeneAll) following the manufacturer’s instructions. The NanoDrop Spectrophotometer (Thermo Fisher Scientific, USA) was then used to quantify the RNA prior to FeCoV detection.

### FeCoV detection by one-step RT-PCR

One-step RT-PCR was used to detect FeCoV by targeting the 3'UTR [21]. One-step RT-PCR was performed in a final volume of 50 µl consisting of 3 µl of template RNA, 25 µl of 2X Reaction Mix, 1 µl of 10 µM forward and reverse primers, 2 µl of SuperScript III RT (Invitrogen, Thermo Fisher, USA), and distilled water. The RT-PCR assay included a cDNA synthesis step at 55°C for 15 min, followed by 94°C for 2 min, and then 40 cycles of 94°C for 15 s, 55°C for 30 s, and 68°C for 1 min, with a final extension at 68°C for 5 min. PCR products were analyzed on a 1.5% agarose gel containing RedSafeTM (iNtRON Biotechnology, Inc., Korea) at 100 V for 45 min. The expected size of the FeCoV-positive amplified products was 223 bp. Positive and no-template controls were used in the RT-PCR reaction to ensure the reliable detection of FeCoV. Laboratory standards were implemented to prevent contamination, including the separation of workspaces for RNA extraction and PCR preparation and the use of sterilized equipment and utensils.

### Genotyping of FeCoV using one-step RT-PCR

All FeCoV-positive samples were genotyped by one-step RT-PCR using primers specific for the *S* gene classification [9, 22]. One-step RT-PCR was performed using SuperScript™ III RT-PCR with Platinum™ Taq Mix (Invitrogen, Thermo Fisher Scientific). For genotyping, the PCR reaction and conditions were similar to those used for virus detection (FeCoV), except for the annealing temperature of 45°C for 30 s. PCR products were run on a 1.5% agarose gel mixed with RedSafeTM (iNtRON Biotechnology, Inc.) at 100 V for 45 min. The samples with 376- and 283-bp amplification products were classified as FeCoV genotypes I and II, respectively.

### Whole-genome and *S* gene sequencing of the FeCoV strain

Four FeCoV-positive samples (n = 4) were subjected to WGS. In addition, complete *S* gene sequencing was performed for six FeCoVs (n = 6). The FeCoV samples were selected to represent different locations, collection dates, clinical histories, and high-quality RNA samples. Sample selection was also based on the quality of PCR amplicons visualized on agarose gels, with stronger, more distinct bands indicating a higher RNA yield. Each viral gene was amplified using newly designed primer sets from the Primer 3 Plus program, as well as previously described primer sets (Supplementary Table 1) [12, 23]. Nucleotide amplification for each gene was performed using one-step RT-PCR with a final total volume of 50 µl. This consisted of 3 µl of template RNA, 25 µl of 2X Reaction Mix, 10 µl of 10 µM forward and reverse primers, 2 µl of SuperScript III RT (Invitrogen), and distilled water. Under these conditions, a cDNA synthesis step was included at 55°C for 30 min, followed by an initial denaturation step at 94°C for 2 min, 40 cycles of denaturation at 94°C for 30 s, annealing at 45°C–48°C for 30 s, and extension at 68°C for 1–4 min, along with a final extension step at 68°C for 5–10 min. Agarose gel electrophoresis was performed to confirm positive PCR amplification. The PCR products were then purified using NucleoSpin® Gel and PCR Clean-up (MACHEREY-NAGELTM, Germany).

**Table 1:** Sample collection and FeCoV detection using RT-PCR

Demographic factors	No. of rectal swabs	FeCoV positive / samples tested (% FeCoV-positive)
Year		
2022	95	27/95 (28.42%)
2023	376	76/376 (20.21%)
Total	471	103/471 (21.87%)
Age of the animals		
< 6 months	123	35/123 (28.46%)
6 months to 2 years	151	30/151 (19.87%)
> 2 years	185	38/185 (20.54%)
Unknown	12	0/12 (0%)
Total	471	103/471 (21.87%)
Sex		
Male	163	39/163 (23.93%)
Neutered male	80	13/80 (16.25%)
Female	158	37/158 (23.42%)
Neutered female	59	12/59 (20.34%)
Unknown	11	2/11 (18.18%)
Total	471	103/471 (21.87%)
Breed		
Pure breed	229	33/229 (14.41%)
Mixed	176	53/176 (30.11%)
Unknown	66	17/66 (25.76%)
Total	471	103/471 (21.87%)
Clinical status		
Asymptomatic	253	60/253 (23.72%)
Symptomatic	218	43/218 (19.72%)
Total	471	103/471 (21.87%)

FeCoV = Feline coronavirus, RT-PCR = Reverse transcription polymerase chain reaction

Oxford Nanopore sequencing technology was used for WGS and *S* gene sequencing. To perform Oxford Nanopore sequencing, the pooled PCR products of each gene were prepared and sequenced using the Oxford Nanopore sequencing device and MinION flow cells with the Oxford Nanopore rapid sequencing kit V14 (SQK-RAD114) according to the rapid sequencing protocol (ONT, UK). For WGS and *S* gene sequencing, the DNA library and the flow cell priming mix were prepared according to the manufacturer’s instructions and sequenced using a sequencing device (MinION Mk1b device) and MinION flow cells (FLO-FLG114, R10.4.1). MinKNOW software (version 24.11.8; ONT, UK) was used for sequencing control and raw signal acquisition. Basecalling was performed using the GPU-enabled Guppy basecaller (version 6.5.7) with a minimum Q-score threshold of 7. The sequencing duration was set to 12 h. The EPI2ME platform (version 5.2.3) was used to identify potential references for sequencing data using the wf-alignment workflow, with the reference set as all FeCoV references in the National Center for Biotechnology Information (NCBI) database. The reads were aligned to the references using Minimap2 (version 2.28). The resulting alignments were then polished using Racon (version 0.5.0), and Medaka (version 2.0.0) was used to refine the consensus sequence. This study employed reference-guided annotation for genome annotation. ORF and gene features were inferred based on the reference genomes of FeCoV-I (UU88; JN183882.1) and FeCoV-II (WSU-79-1683; JN634064) and subsequently confirmed by nucleotide Basic Local Alignment Search Tool (BLAST) searches against the NCBI database (Supplementary Table 2). The nucleotide sequences of each *FeCoV* gene segment were retrieved in a FASTA file format and compared to the NCBI database using BLAST to identify the closest matching reference sequence for each gene segment. Finally, the consensus sequence of each viral gene segment was exported in the FASTA format for further analysis.

**Table 2:** FeCoV occurrence and association of FeCoVs by age, clinical status, and season

Factors	FeCoV			

	Positive (%)	Negative (%)	Chi-square	P-value
Age				
Up to 6 months	35 (28.46)	88 (71.54)	7.03	0.68
Older than 6 months to 2 years	30 (19.87)	121 (80.13)		
More than 2 years	38 (20.54)	147 (79.46)		
Unknown	0 (0.00)	12 (100.00)		
Total	103	368		
Clinical Status				
Asymptomatic	60 (23.72)	193 (76.28)	1.09	0.29
Symptomatic	43 (19.72)	175 (80.28)		
Total	103	368		
Season				
Winter (November–January)	31 (24.03)	98 (75.97)	1.31	0.52
Summer (February– May)	38 (23.17)	126 (76.83)		
Rainy (June–October)	34 (19.10)	144 (80.90)		
Total	103	368		

* Statistical significance = p < 0.05, FeCoV = Feline coronavirus

### Phylogenetic and genetic analysis of the *FeCoV* gene

Phylogenetic analysis of the S gene and whole-genome sequences (WGS) of FeCoV was performed by comparing the nucleotide sequences of Thai FeCoVs with reference CoVs from the coronavirus family available in the GenBank database, representing various geographical, host, and species origins. The coronavirus reference sequences from the *Alphacoronavirus, Betacoronavirus, Gammacoronavirus*, and *Deltacoronavirus* genera were used to root the trees and differentiate the host origin and coronavirus species. Phylogenetic trees of the *S* gene and WGS of FeCoVs were constructed using Molecular Evolutionary Genetics Analysis (MEGA) version 11.0 (Tempe, AZ, USA) with the neighbor-joining method applying the Kimura 2-parameter and 1,000 bootstrap replicates [24]. The Kimura 2-parameter (K2P) model was chosen because of its ability to effectively account for transition/transversion rate differences and provide reliable phylogenetic analysis of similar viral sequences. A pairwise comparison of the nucleotides and amino acids of FeCoV was conducted with FeCoVs from both the same and different genotypes. Genetic analysis was performed by aligning and comparing the *S* gene amino acid sequences of FeCoV with those of reference FeCoVs from the same and different genotypes using MegAlign software V.5.03 (DNASTAR Inc., Wisconsin, USA). We evaluated the unique and variable amino acids associated with receptor binding and viral fusion for cell entry, as well as host preferences.

### Statistical analysis

The association between FeCoV occurrence in domestic cats and factors such as animal age, season, and clinical status was analyzed using the chi-square test (SPSS Statistics, version 29.0.1.0, IBM Corp., NY, USA). A p-value of < 0.05 was considered statistically significant. Categorical variables, including age, were classified based on animals; clinical status was grouped as symptomatic or asymptomatic; and season was categorized according to Thailand’s climate pattern. These groupings ensured adequate sample sizes per category and facilitated meaningful comparisons. The assumptions for chi-square testing were verified prior to analysis, including the assumption of categorical data, independent observations, and expected frequencies in contingency table cells that were generally greater than 5.

## RESULTS

### FeCoV occurrence in domestic cats

We conducted a cross-sectional survey of FeCoV in domestic cats (n = 471) at private small animal hospitals in Bangkok, Nonthaburi, and Samut Prakan, Thailand, from October 2022 to October 2023. FeCoV positivity was 21.87% (103/471) in domestic cats (Table 1). The FeCoV positivity was 28.42% (27/95) in 2022 and 20.21% (76/376) in 2023. By age, we found the highest FeCoV detection in younger cats (up to 6 months) at 28.46% (35/123), but this was not statistically significant. FeCoVs were most frequently detected in the winter (November to January) (24.03%) and summer (February to May) (23.17%) seasons, but no statistical significance was observed. By clinical status, FeCoV can be detected in both symptomatic and asymptomatic cats, with the highest positivity in asymptomatic cats (23.72%), but this difference was not statistically significant (Table 2). FeCoV genotype I (99.03%, 102/103) was more prevalent than FeCoV genotype II (0.97%, 1/103) (Table 3).

**Table 3:** Genotyping of FeCoV-positive samples in cats

Virus	Year	Genotype			Total

		I	IIa	Mixed (I + IIa)	
FeCoV	2022	27	0	0	27
	2023	75	1	0	76
Total		103	1	0	103

FeCoV = Feline coronavirus

### Whole-genome and *S* gene sequencing output

The WGS of FeCoV (n = 4) were successfully sequenced for phylogenetic and genetic analysis. The complete *S* gene sequences of FeCoV (n = 6) were also available. All Thai FeCoV nucleotide sequences were submitted to the database under the GenBank accession numbers (PV797400–PV797409). Table 4 and Supplemental Tables 2 and 3 provide detailed information on the FeCoVs characterized in this study.

**Table 4:** Detailed information on FeCoV characterized from cats

No.	ID	Year	Location	Age	Sex	Breed	Clinical sign	Gene sequencing		Accession No.

								Genotype I	Genotype IIa	
1	CU30655	Oct-2022	Bangkok	2 months	F	Scottish Fold	Healthy	S		PV797404
2	CU30743	Nov-2022	Bangkok	7 years	F	Domestic Short Hair	N/A	WGS		PV797400
3	CU31310	Dec-2022	Bangkok	2 years 5 months	NF	British Short Hair	Diarrhea	WGS		PV797401
4	CU31327	Dec-2022	Bangkok	4 months	F	N/A	Healthy	S		PV797405
5	CU32238	Mar-2023	Bangkok	5 months	F	Domestic Short Hair	Diarrhea	S		PV797406
6	CU32640	May-2023	Nonthaburi	2 months	F	Domestic Short Hair	Diarrhea	S		PV797407
7	CU32825	June-2023	Nonthaburi	1 year	F	Domestic Short Hair	Diarrhea		WGS	PV797402
8	CU33011	June-2023	Nonthaburi	25 days	M	British Short Hair	Diarrhea	S		PV797408
9	CU33180	July-2023	Nonthaburi	12 days	F	Domestic Short Hair	Diarrhea	WGS		PV797403
10	CU33441	Aug-2023	Nonthaburi	1 year 6 months	F	Scottish Fold	Diarrhea	S		PV797409

N/A=not available, FeCoV = Feline corona virus, WGS = Whole genome sequence

In the phylogenetic analysis of the *S* gene, one FeCoV-I strain (CU33441) was closely related to Chinese FeCoV-I (HLJ/DQ/2016/01; SMU-CD60). Another Thai FeCoV-I strain (CU31327) was closely related to FeCoV-I from China (HLJ/HRB/2016/13). Thai FeCoV-I (CU33011) was closely related to US-derived FeCoV-I (UCD1). The other six Thai FeCoV-I strains were closely related to FeCoV-I from the Netherlands (UU10 and UU11). The Thai FeCoV-II strain (CU32825) was closely related to the Chinese FeCoV-II strain (ZJU1617; SMU-CD59). Additionally, all Thai FeCoV genotypes I and II were grouped with FeCoVs described as FeCoV biotypes from other countries (Figure 2).

### Phylogenetic relationships based on whole-genome and *S* gene analysis

**Figure 1:**
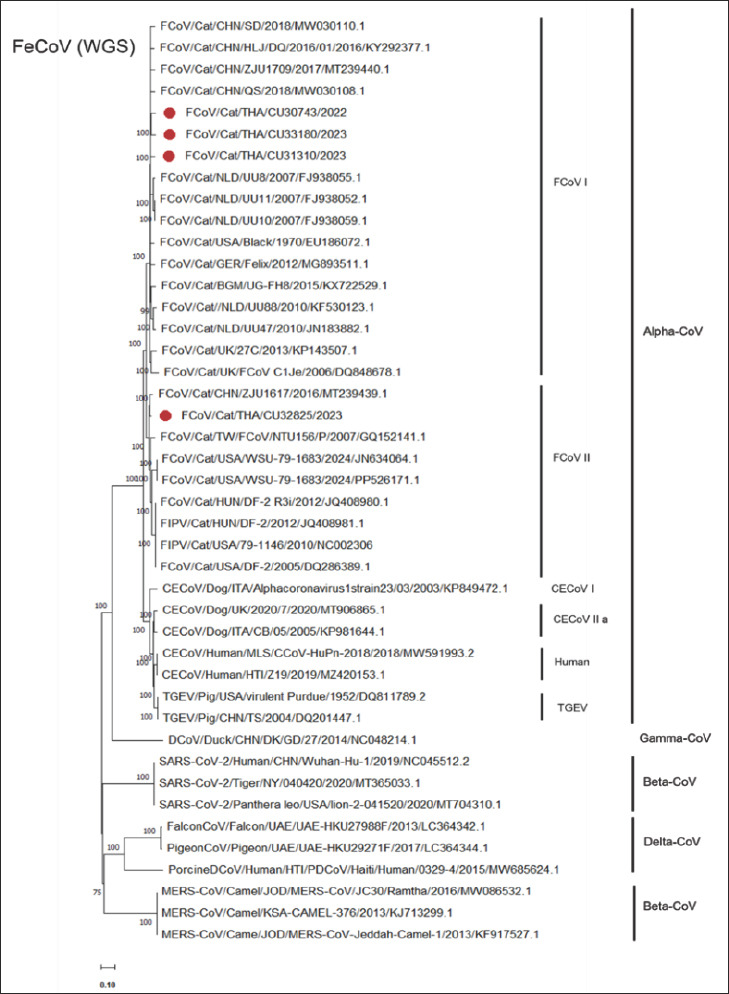
Phylogenetic tree of whole-genome sequences of Thai feline coronavirus (FeCoV) using the neighboring method with the Kimura-2 model and 1,000 bootstrapping replicates. The red circle represents the Thai FeCoV.

The phylogenetic analysis of whole-genome sequences and complete *S* gene of FeCoVs showed that all Thai FeCoV genotype I (FeCoV-I) were closely related to FeCoV-I from China (ZJU1709; QS; HLJ/DQ/2016/01) and the Netherlands (UU8; UU11; UU10). Similarly, Thai FeCoV-II (CU32825) was closely related to Chinese-FeCoV-II (ZJU1617) (Figure 1).

**Figure 2:**
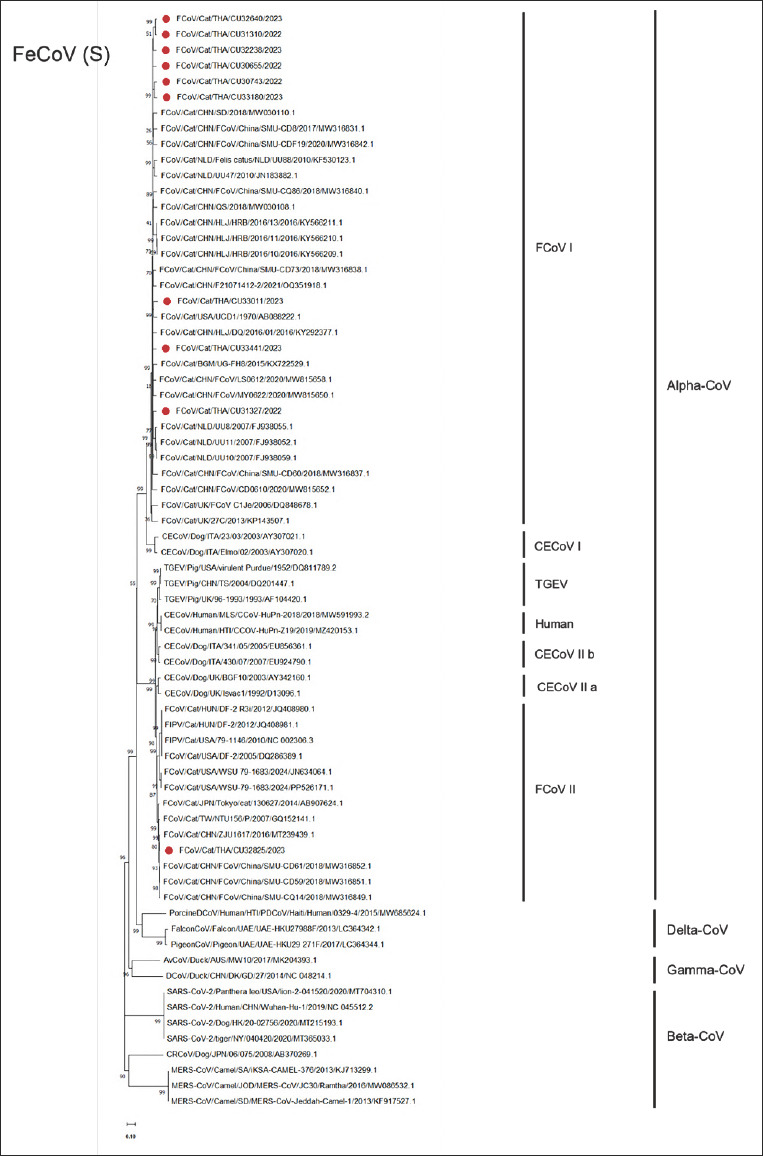
Phylogenetic tree of S gene nucleotide sequences of Thai feline coronavirus (FeCoV) using the neighboring method with the Kimura-2 model and 1,000 bootstrapping replicates. The red circle represents the Thai FeCoV.

### Pairwise nucleotide and amino acid identity analysis

Pairwise comparison of the WGS of the Thai FeCoV-I (CU33180) showed high nucleotide (nt%, 87.8% and 92.3%) and amino acid identities (aa%; 84.5% and 88.2%), respectively, compared with the other Thai FeCoV-I, CU31310 and CU30743. Thai FeCoV-I (CU33180) has a high degree of similarity to FeCoV-I from China (SD) (91.6% nt; 93.1% aa identities). Similarly, the WGS of the Thai FeCoV-I (CU33180) showed high nucleotide (% nt identities, 90.9% – 91.6%) and amino acid (% aa identities, 87.3% – 92.8%) when compared to other reference FeCoV-I (Table 5). The Thai FeCoV-II (CU32825) showed high nucleotide (95.6%) and amino acid (93.8%) identities with the Chinese FeCoV-II (ZJU1617) (Table 6). The WGS of the Thai FeCoV-II (CU32825) showed low nucleotide identities (nt%, 78.4% – 83.9%) and amino acid identities (aa%; 74.0% – 80.3%) compared to the Thai FeCoV-Is (Table 6).

**Table 5:** Nucleotide (nt) and amino acid (aa) identities of the whole Thai FeCoV-I (CU33180) genome with reference CoVs

Strain	Accession No.	Genotype	Spp.	Country	Year	WGS	ORF1ab	S	3a	3b	3c	E	M	N	7a	7b

						n (aa%)	n (aa%)	n (aa%)	n (aa%)	n (aa%)	n (aa%)	n (aa%)	n (aa%)	n (aa%)	n (aa%)	n (aa%)
CU33180	PV797403	FeCoV-I	Feline	Thailand	2023	100.0 (100.0)	100.0 (100.0)	100.0 (100.0)	100.0 (100.0)	100.0 (100.0)	100.0 (100.0)	100.0 (100.0)	100.0 (100.0)	100.0 (100.0)	100.0 (100.0)	100.0 (100.0)
CU30655	PV797404	FeCoV-I	Feline	Thailand	2022	(-)	(-)	86.5 (91.6)	(-)	(-)	(-)	(-)	(-)	(-)	(-)	(-)
CU30743	PV797400	FeCoV-I	Feline	Thailand	2022	92.3 (88.2)	92.4 (89.2)	90.8 (92.2)	93.5 (93.5)	92.9 (93.1)	97.4 (97.7)	90.3 (92.1)	89.7 (93.2)	94.0 (92.1)	93.3 (92.0)	90.8 (87.0)
CU31310	PV797401	FeCoV-I	Feline	Thailand	2023	87.8 (84.5)	92.2 (90.4)	87.4 (91.7)	94.6 (95.2)	92.9 (93.1)	95.8 (96.5)	93.0 (97.4)	92.0 (93.6)	92.3 (92.4)	95.2 (93.4)	90.1 (91.5)
CU31327	PV797405	FeCoV-I	Feline	Thailand	2023	(-)	(-)	85.5 (90.1)	(-)	(-)	(-)	(-)	(-)	(-)	(-)	(-)
CU32238	PV797406	FeCoV-I	Feline	Thailand	2023	(-)	(-)	87.0 (92.3)	(-)	(-)	(-)	(-)	(-)	(-)	(-)	(-)
CU32640	PV797407	FeCoV-I	Feline	Thailand	2023	(-)	(-)	86.0 (91.0)	(-)	(-)	(-)	(-)	(-)	(-)	(-)	(-)
CU33011	PV797408	FeCoV-I	Feline	Thailand	2023	(-)	(-)	87.3 (91.7)	(-)	(-)	(-)	(-)	(-)	(-)	(-)	(-)
CU33441	PV797409	FeCoV-I	Feline	Thailand	2023	(-)	(-)	84.9 (89.9)	(-)	(-)	(-)	(-)	(-)	(-)	(-)	(-)
CU32825	PV797402	FeCoV-II	Feline	Thailand	2023	78.4 (74.5)	92.1 (89.5)	31.5 (28.4)	60.1 (64.8)	53.4 (59.5)	85.1 (75.7)	92.6 (96.1)	92.3 (92.4)	94.5 (95.7)	95.6 (93.4)	94.3 (94.1)

**FeCoV reference strains**																

SD	MW030110.1	FeCoV-I	Feline	China	2018	91.6 (93.1)	92.4 (90.8)	86.4 (92.3)	94.6 (95.2)	92.9 (93.1)	95.8 (96.5)	91.7 (98.7)	90.6 (93.2)	92.8 (93.9)	95.6 (94.7)	90.1 (88.8)
SMU-CD86	MW316840.1	FeCoV-I	Feline	China	2020	NA	NA	87.5 (92.4)	NA	NA	NA	NA	NA	NA	NA	NA
UCD1	AB0882222.1	FeCoV-I	Feline	USA	1970	NA	NA	84.8 (90.1)	NA	NA	NA	NA	NA	NA	NA	NA
HLJ/DQ/2016/01	KY292377.1	FeCoV-I	Feline	China	2016	91.0 (92.8)	92.0 (90.2)	84.9 (91.3)	93.5 (93.5)	92.0 (92.2)	95.9 (96.5)	91.7 (97.4)	91.2 (94.0)	91.8 (91.2)	95.2 (94.7)	90.7 (88.8)
UG-FH8	KX722529.1	FeCoV-I	Feline	Belgium	2105	90.9 (87.3)	91.8 (90.1)	84.3 (90.1)	94.1 (95.2)	95.7 (95.7)	94.7 (96.5)	92.6 (97.4)	91.4 (93.2)	91.7 (93.0)	92.0 (90.6)	89.8 (89.7)
ZJU1617	MT239439.1	FeCoV-II	Feline	China	2016	77.4 (72.8)	91.0 (87.8)	30.8 (27.6)	60.1 (64.8)	53.4 (59.5)	84.2 (74.2)	90.7 (92.1)	89.7 (92.0)	92.7 (93.0)	95.6 (93.4)	92.6 (91.5)
NTU156/P	GQ152141.1	FeCoV-II	Feline	Taiwan	2007	75.9 (70.0)	89.4 (84.8)	31.4 (28.3)	62.0 (67.0)	58.4 (63.1)	47.1 (32.9)	78.0 (77.4)	83.5 (86.8)	90.6 (91.2)	95.2 (94.7)	93.2 (93.3)
SMU-CD14	MW316852.1	FeCoV-II	Feline	China	2018	NA	NA	30.5 (27.1)	NA	NA	NA	NA	NA	NA	NA	NA
SMU-CD59	MW31685.1	FeCoV-II	Feline	China	2018	NA	NA	30.9 (28.6)	NA	NA	NA	NA	NA	NA	NA	NA

**Alphacoronavirus 1 reference strains were used**																

Alphacoronavirus 1 strain 23/03	KP849472.1	CECoV I	Canine	Italy	2003	62.0 (58.2)	81.0 (75.8)	29.6 (27.4)	68.0 (67.0)	68.0 (71.2)	84.2 (75.7)	80.8 (80.5)	80.5 (85.0)	75.9 (72.3)	88.5 (86.3)	61.3 (51.9)
CB/05	KP981644.1	CECoV II	Canine	Italy	2005	70.9 (64.2)	80.8 (75.8)	32.0 (29.8)	63.0 (64.8)	49.6 (40.1)	81.7 (77.1)	79.7 (77.4)	78.6 (82.8)	74.8 (74.5)	82.7 (80.2)	60.4 (53.2)
CCoV-HuPn-2018	MW591993.2	CECoV II	Human	Malaysia	2018	67.7 (61.2)	80.4 (75.3)	30.9 (27.1)	63.0 (64.8)	50.6 (58.2)	83.2 (77.1)	79.2 (75.8)	78.4 (80.9)	74.6 (74.2)	82.2 (77.0)	60.1 (56.0)
Z19	MZ420153.1	CECoV II	Human	Hati	2019	67.8 (61.4)	80.4 (75.4)	30.9 (26.9)	63.0 (64.8)	50.6 (58.2)	83.2 (77.1)	76.8 (75.8)	79.3 (82.8)	74.6 (75.3)	78.0 (77.0)	60.7 (55.7)
virulent Purdue	DQ811789.2	TGEV	Pig	USA	1952	67.2 (60.9)	80.3 (75.4)	30.4 (26.8)	61.3 (64.8)	N/A	81.3 (74.2)	78.0 (74.1)	77.2 (81.8)	73.4 (73.5)	78.6 (77.0)	NA
TS	DQ201447.1	TGEV	Pig	China	2004	66.9 (60.2)	80.0 (74.6)	29.9 (26.4)	57.3 (60.2)	N/A	80.4 (72.7)	78.0 (74.1)	76.8 (81.4)	72.9 (73.8)	76.8 (72.1)	NA

N/A = not available, FeCoV = Feline corona virus

100%, INLINE 99 ~ 95%, INLINE 94 ~ 90%, INLINE < 90% INLINE

**Table 6:** Nucleotide (nt) and amino acid (aa) identities of the whole Thai FeCoV-II (CU32825) genome with reference CoVs.

Strain	Accession No.	Genotype	Spp.	Country	Year	WGS	ORF1ab	S	3a	3b	3c	E	M	N	7a	7b

						n (aa%)	n (aa%)	n (aa%)	n (aa%)	n (aa%)	n (aa%)	n (aa%)	n (aa%)	n (aa%)	n (aa%)	n (aa%)
CU32825	PV797402	FeCoV-II	Feline	Thailand	2023	100.0 (100.0)	100.0 (100.0)	100.0 (100.0)	100.0 (100.0)	100.0 (100.0)	100.0 (100.0)	100.0 (100.0)	100.0 (100.0)	100.0 (100.0)	100.0 (100.0)	100.0 (100.0)
CU30655	PV797404	FeCoV-I	Feline	Thailand	2022	(-)	(-)	30.6 (28.8)	(-)	(-)	(-)	(-)	(-)	(-)	(-)	(-)
CU30743	PV797400	FeCoV-I	Feline	Thailand	2022	78.8 (74.0)	92.4 (88.8)	32.2 (28.6)	56.4 (64.8)	59.2 (63.1)	83.2 (74.2)	91.3 (03.5)	92.0 (94.0)	95.1 (95.1)	95.2 (96.1)	92.7 (89.7)
CU31310	PV797401	FeCoV-I	Feline	Thailand	2023	83.9 (80.3)	92.9 (90.5)	32.5 (29.3)	63.0 (64.8)	59.2 (63.1)	83.7 (74.2)	94.4 (98.7)	92.7 (94.4)	94.9 (95.7)	96.1 (97.4)	92.1 (93.3)
CU31327	PV797405	FeCoV-I	Feline	Thailand	2023	(-)	(-)	32.6 (28.4)	(-)	(-)	(-)	(-)	(-)	(-)	(-)	(-)
CU32238	PV797406	FeCoV-I	Feline	Thailand	2023	(-)	(-)	32.1 (29.3)	(-)	(-)	(-)	(-)	(-)	(-)	(-)	(-)
CU32640	PV797407	FeCoV-I	Feline	Thailand	2023	(-)	(-)	31.4 (28.9)	(-)	(-)	(-)	(-)	(-)	(-)	(-)	(-)
CU33011	PV797408	FeCoV-I	Feline	Thailand	2023	(-)	(-)	32.0 (28.3)	(-)	(-)	(-)	(-)	(-)	(-)	(-)	(-)
CU33180	PV797403	FeCoV-I	Feline	Thailand	2023	78.4 (74.5)	92.1 (89.5)	31.5 (28.4)	60.1 (64.8)	53.4 (59.5)	85.1 (74.2)	92.6 (96.1)	92.3 (92.4)	94.5 (95.7)	95.6 (93.4)	94.3 (94.1)
CU33441	PV797409	FeCoV-I	Feline	Thailand	2023	(-)	(-)	32.6 (27.6)	(-)	(-)	(-)	(-)	(-)	(-)	(-)	(-)
Reference FeCoV																
SD	MW030110.1	FeCoV-I	Feline	China	2018	79.1 (75.7)	92.9 (90.8)	32.0 (28.6)	63.0 (64.8)	59.2 (63.1)	83.7 (74.2)	92.6 (96.1)	92.0 (94.0)	96.0 (97.4)	96.5 (98.7)	91.6 (90.6)
SMU-CD86	MW316840.1	FeCoV-I	Feline	China	2020	NA	NA	31.8 (27.8)	NA	NA	NA	NA	NA	NA	NA	NA
UCD1	AB0882222.1	FeCoV-I	Feline	USA	1970	NA	NA	30.9 (27.2)	NA	NA	NA	NA	NA	NA	NA	NA
HLJ/DQ/2016/01	KY292377.1	FeCoV-I	Feline	China	2016	79.2 (75.8)	93.0 (90.6)	31.8 (28.4)	57.4 (64.8)	59.5 (63.1)	83.8 (74.2)	94.9 (98.7)	93.1 (94.9)	95.1 (94.2)	96.1 (98.7)	94.1 (91.5)
UG-FH8	KX722529.1	FeCoV-I	Feline	Belgium	2105	78.7 (75.4)	92.3 (90.0)	32.1 (27.7)	59.3 (67.0)	58.7 (63.1)	85.6 (74.2)	94.0 (98.7)	93.2 (92.8)	94.7 (96.0)	95.7 (97.4)	90.4 (89.7)
ZJU1617	MT239439.1	FeCoV-II	Feline	China	2016	95.6 (93.8)	95.0 (93.5)	98.0 (97.7)	100.0 (100.0)	100.0 (100.0)	99.3 (98.9)	93.6 (96.1)	95.5 (96.9)	96.1 (96.9)	98.3 (100.0)	96.4 (95.0)
NTU156/P	GQ152141.1	FeCoV-II	Feline	Taiwan	2007	92.4 (92.1)	93.2 (92.5)	95.9 (97.2)	98.4 (98.4)	97.5 (97.5)	65.3 (63.4)	79.7 (78.9)	87.2 (89.8)	94.0 (94.2)	97.8 (98.7)	94.6 (93.3)
SMU-CD14	MW316852.1	FeCoV-II	Feline	China	2018	NA	NA	98.1 (98.9)	NA	NA	NA	NA	NA	NA	NA	NA
SMU-CD59	MW31685.1	FeCoV-II	Feline	China	2018	NA	NA	97.1 (97.9)	NA	NA	NA	NA	NA	NA	NA	NA
**Alphacoronavirus 1 reference strains were used**																
Alphacoronavirus 1 strain 23/03	KP849472.1	CECoV I	Canine	Italy	2003	65.3 (63.9)	83.2 (80.2)	80. 2. (81.1)	86.8 (91.9)	73.6 (75.6)	90.7 (87.9)	82.4 (82.0)	82.1 (85.5)	76.9 (73.8)	88.0 (86.3)	61.7 (51.9)
CB/05	KP981644.1	CECoV II	Canine	Italy	2005	78.1 (73.4)	83.2 (80.3)	89.8 (91.8)	95.7 (95.2)	24.7 (29.0)	93.8 (95.3)	81.4 (78.9)	80.1 (83.2)	75.8 (75.3)	83.3 (80.2)	62.7 (54.5)
CCoV-HuPn-2018	MW591993.2	CECoV II	Human	Malaysia	2018	82.1 (78.2)	83.0 (80.2)	81.1 (82.8)	95.7 (96.8)	91.2 (91.3)	94.3 (95.3)	80.8 (77.4)	81.3 (84.6)	76.0 (75.3)	81.6 (77.0)	68.7 (58.9)
Z19	MZ420153.1	CECoV II	Human	Hati	2019	82.5 (78.4)	83.1 (80.2)	81.2 (82.9)	95.7 (96.8)	91.2 (91.3)	94.3 (95.3)	78.5 (77.4)	82.2 (84.1)	75.8 (76.0)	78.6 (77.0)	63.8 (57.0)
Virulent Purdue	DQ811789.2	TGEV	Pig	USA	1952	76.5 (72.3)	82.8 (80.0)	80.8 (81.2)	94.6 (95.2)	N/A	93.1 (92.9)	80.8 (75.8)	80.5 (84.6)	74.3 (73.5)	79.2 (77.0)	NA
TS	DQ201447.1	TGEV	Pig	China	2004	76.5 (71.6)	82.5 (79.2)	80.1 (80.3)	92.2 (88.4)	N/A	92.2 (91.7)	80.8 (75.8)	80.1 (84.6)	74.2 (74.5)	77.5 (72.1)	NA

N/A = not available, FeCoV = Feline corona virus

100%, INLINE 99 ~ 95%, INLINE 94 ~ 90%, INLINE < 90% INLINE

### Genetic characterization of S gene regions

For the genetic analysis of FeCoVs, the deduced amino acids of the *S* gene of Thai FeCoVs were aligned to those of reference FeCoVs from the same and different genotypes. Our results showed that the amino acids in the S1/S2 and S2 regions of Thai FeCoV-I and reference strains were identical (no mutation). Thai FeCoV-II (CU32825) contained a six-amino-acid deletion (positions 828–830) at the S1/S2 cleavage site and a three-amino-acid insertion (positions 1036–1038) at the S2 (Table 7).

**Table 7:** Genetic analysis of the amino acid sequence of the *S* gene of Thai FeCoV compared with reference FeCoVs.

Virus	Country	Year	Genotype	Putative Pathotypes	Predicted proteolytic cleavage sites	

					S1/S2 (828 - 830)	S2 (1034 - 1039)
Ref sequences used in this study						
HLJ/DQ/2016/01	China	2018	I	FeCoV	RRSRRS	KR---S
SD	China	2018	I	FeCoV	RRSRRS	KR---S
FCoV/CD0610	China	2020	I	FeCoV	RRARRS	KR---S
USD1	USA	1970	I	FeCoV	RRSRGS	QR---S
UG-FH8	Belgium	2015	I	FeCoV	KRLRRS	KR---S
UU10	Netherlands	2007	I	FeCoV	KRSRRS	KR---S
UU11	Netherlands	2007	I	FeCoV	KRSRRS	KR---S
UU34	Netherlands	2007	I	FeCoV	RRSRRS	KR---S
UU8	Netherlands	2007	I	FIPV	RRSRRS	KR---S
HLJ/HRB/2016/11	China	2016	I	FIPV	RRSRRS	KR---S
HLJ/HRB/2016/10	China	2016	I	FIPV	RRSRRS	KR---S
HLJ/HRB/2016/13	China	2016	I	FIPV	RRSRRS	KR---S
QS	China	2018	I	FIPV	RRSRTS	KR---S
BLACK	USA	1970	I	FIPV	KRSRRP	VR---S
26M	UK	2013	I	FIPV	RGARRS	KR---S
FCoVCC1Je	UK	2006	I	FIPV	RQSRRS	KR---S
79-1146	USA	2010	II	FIPV	DEL	KRKYGS
DF-2	Hungary	2012	II	FIPV	DEL	KRKYGS
DF-2	USA	2005	II	FeCoV	DEL	KRKYGS
DF-2R3i	Hungary	2012	II	FeCoV	DEL	KRKYGS
WSU79-1683	USA	2024	II	FeCoV	DEL	KRKYRS
Tokyo/cat/130627	Japan	2014	II	FeCoV	DEL	KRKYRS
NTU156/P	Taiwan	2007	II	FeCoV	DEL	KRKYRS
SMU-CQ14	China	2018	II	FeCoV	DEL	KRKYRS
SMU-CQ59	China	2018	II	FeCoV	DEL	KRKYRS

This study						
THA/CU30655	Thailand	2022	I	FeCoV	RRSRRS	KR---S
THA/CU30743	Thailand	2022	I	FeCoV	RRSRRS	KR---S
THA/CU31310	Thailand	2023	I	FeCoV	RRSRRS	KR---S
THA/CU31327	Thailand	2023	I	FeCoV	RRSRRS	KR---S
THA/CU32238	Thailand	2023	I	FeCoV	RRSRRS	KR---S
THA/CU32640	Thailand	2023	I	FeCoV	RRSRRS	KR---S
THA/CU33011	Thailand	2023	I	FeCoV	RRARRS	KR---S
THA/CU33180	Thailand	2023	I	FeCoV	RRSRRS	KR---S
THA/CU33441	Thailand	2023	I	FeCoV	RRSRRS	KR---S
THA/CU32825	Thailand	2023	II	FeCoV	DEL	KRKYRS

FeCoV = Feline coronavirus

## DISCUSSION

### Occurrence of FeCoV in domestic cats

In this study, the occurrence of FeCoV in domestic cats in Bangkok and its vicinity from October 2022 to October 2023 was 21.87%. This occurrence is lower than those reported in earlier studies in Thailand, where FeCoV was found at 30.97% (25). This discrepancy might be due to differences in sample size, geographic factors, and improvements in infection control measures. Our results showed that FeCoVs were highly detected in young cats (up to 6 months of age). This observation, in agreement with previous reports from Australia, China, and Japan, demonstrated that a younger age is a significant factor in FeCoV positivity (26–28). Similarly, our findings were consistent with the current report of FeCoVs in Thailand (12). In contrast, other studies from Australia, Hungary, and Malaysia reported that FeCoV infection was not associated with the age of the cat (26,29). The susceptibility and resistance to FeCoV associated with age remain unknown and vary across different geographic locations, as indicated by Li *et al*. (28).

### Seasonal and clinical distribution of FeCoV

The highest positivity for FeCoV was found in summer and winter, compared to the rainy seasons. This finding was consistent with previous studies in Korea and Thailand, which found that FeCoV was the most prevalent during winter [12, 30]. However, some studies have reported that FeCoV is ubiquitous and mostly subclinical in all cats, occurring consistently throughout the year without any seasonal variation [30]. In this study, a high FeCoV positivity rate was observed among asymptomatic cats, consistent with previous studies from Thailand [9, 12]. The results suggested that asymptomatic cats may serve as potential reservoirs for FeCoV, contributing to viral transmission to susceptible populations. This consistent association between FeCoV infection and the health status of cats in Thailand highlights the importance of including asymptomatic cats in the development of effective prevention and control strategies for FeCoV infection in feline populations. Additionally, the proportion of FeCoV-infected cats that develop clinical signs, such as diarrhea, remains unclear [31].

### Distribution and implications of FeCoV genotypes

Regarding genotyping results, FeCoV genotype I was identified as the predominant genotype in Thai cat populations. Other studies from Europe and the Americas reported a high prevalence of FeCoV-I, ranging from 80% to 95%, while FeCoV-II is less common [32]. This finding suggested that FeCoV-I may circulate more widely, possibly because current vaccines target only the FeCoV genotype II [33]. The prevalence of FeCoV-I in Thailand has implications for the development of epidemiological and translational vaccines. Most currently available vaccines are based on FeCoV-II strains, which may limit their effectiveness in regions where FeCoV-I is dominant. The high prevalence of FeCoV-I highlights the need for vaccine strategies that incorporate FeCoV-I epitopes or develop broadly protective formulations for both genotypes. From an epidemiological perspective, given the predominance of FeCoV-I, surveillance and diagnostic assays should prioritize It to avoid underestimating infection rates. Continued monitoring of genotype distribution is essential to detect potential shifts in FeCoV genotype prevalence, which could impact both control strategies and vaccine design in Thailand and the region.

### Phylogenetic and molecular characterization of Thai FeCoVs

Phylogenetic analysis of whole-genome sequences and the S gene revealed that all Thai FeCoV genotypes I and II were closely related to FeCoV-I and FeCoV-II. Moreover, all Thai FeCoVs were grouped with all reference FeCoV biotypes, suggesting that Thai FeCoVs could be classified as FeCoV biotypes. This finding highlights the predominant biotypes of FeCoV infection in Thailand’s cat populations. In the S gene, Thai FeCoV-I contained no amino acid mutations in the S1/S2 or S2 regions compared to reference FeCoVs. Similarly, Thai FeCoV-IIs were consistent with reference FeCoV-IIs. Notably, the highly conserved regions in the S1/S2 region could be used to facilitate molecular diagnostic assays, vaccine development, and differentiation between FeCoV and FIPV biotypes. However, the association between pathogenicity and insertion/deletion at the S1/S2 and S2 sites remains unclear. These changes could impact the pathogenicity of FeCoVs (both FeCoV and FIPV biotypes). For example, FeCoVs consist of an S1/S2 cleavage site with the R-R-S-R-R-S motif, whereas FIPV exhibits several substitutions at this site [34, 35]. Thai FeCoVs were highly conserved (no amino acid change) at the conserved furin cleavage motif (R-R-S/A-R-R-S), which is typical for FeCoVs. These findings were consistent with those of a recent study from Vietnam [19]. Additionally, Thai FeCoVs were detected in fecal samples from non-FIP cats, and no clinical cases of FIP were observed during the sampling period. Therefore, the conserved amino acid changes and subclinical presentation suggest that Thai FeCoVs are the predominant FeCoV biotype.

### Genomic features and recombination insights of FeCoV-II

This study revealed that the Thai FeCoV-II exhibited unique genomic features, but there was no evidence of recombination with other coronaviruses from different hosts. It has been reported that FeCoV-II originated from homologous recombination between FeCoV-I and canine coronavirus (CCoV), in which the common recombination hotspots are at the S gene and open reading frame 1ab (ORF1ab) regions [4, 36]. Pairwise comparison of the WGS of the Thai FeCoV-II showed high nucleotide and amino acid identities with FeCoV-II reference strains, but lower nucleotide and amino acid identities to FeCoV-I reference strains and Canine CoVs from dogs. Similarly, phylogenetic analysis of both the WGS and S genes revealed that Thai FeCoV-II was grouped within the FeCoV-II cluster and clearly distinct from other coronaviruses from different hosts. Although recombination analysis (e.g., with SimPlot or RDP4) was not conducted in this study, the phylogenetic analysis of Thai FeCoV-II showed no evidence of recent recombination with other coronaviruses. Future studies should apply recombination detection tools to confirm potential recombination hotspots, particularly in the S gene and ORF1ab, and further clarify the evolutionary history of FeCoV-II in Thailand. In addition, cross-species investigations should be conducted, particularly involving canine coronavirus (CCoV), to understand the recombination events and the potential for spillover between cats and dogs in Thailand.

### Study limitations

This study has some limitations, including time constraints that restricted study sites, sample collection, and clinical follow-up. Moreover, only few FeCoVs were sequenced, which may underrepresent the genetic diversity of circulating FeCoVs in Thailand. Potential confounding factors, such as clinic-level clustering, owner socioeconomic status, and housing conditions, were not assessed due to limited information available in the outpatient data and from pet owners. These factors influence viral transmission and may explain the variability in the occurrence of FeCoV. Additionally, the statistical analysis was limited to bivariate comparisons of FeCoV occurrence with individual categorical factors (age, clinical status, and season), yielding no statistically significant results. Therefore, corrections for multiple testing were not applied. Despite these limitations, this study provided the first FeCoV-II WGS in Thailand and established valuable baseline data for future multi-regional and longitudinal investigations of FeCoVs.

## CONCLUSION

This study provides the most updated molecular epidemiological overview of FeCoV circulating in domestic cats in Bangkok and surrounding provinces, with an overall FeCoV occurrence of 21.87%, the predominance of FeCoV genotype I (99.03%), and the detection of only one FeCoV-II strain. Younger cats (≤6 months) showed the highest positivity, and FeCoV was detected in both summer and winter, although none of the factors showed statistical significance. Importantly, this study generated the first FeCoV-II whole-genome sequence from Thailand, along with whole-genome and complete *S gene* sequences for multiple FeCoV-I strains. Phylogenetic analysis demonstrated that Thai FeCoV-I strains were closely related to those from China, the Netherlands, and the United States, while the Thai FeCoV-II clustered tightly with Chinese FeCoV-II. Genetic characterization revealed high conservation in the S1/S2 and S2 cleavage regions among Thai FeCoVs, consistent with previously described FeCoV biotypes, suggesting circulation of predominantly low-virulence strains in the sampled population.

The findings have several practical implications. First, the dominance of FeCoV-I highlights the urgent need to reconsider current vaccine formulations, which are largely based on FeCoV-II strains and may not provide optimal protection in regions where FeCoV-I prevails. Second, the high positivity rate in asymptomatic cats underscores their potential as silent reservoirs, underscoring the importance of routine surveillance, improved outbreak monitoring, and targeted prevention strategies. The genomic data generated in this study also enhance diagnostic assay design, particularly by identifying conserved spike regions relevant for molecular differentiation between FeCoV and FIPV.

A key strength of this study is the integration of epidemiological data with whole-genome and S gene sequencing, enabling a more comprehensive understanding of circulating FeCoV genotypes and their evolutionary relationships. Additionally, the multi-hospital sampling approach improved representativeness across the Bangkok metropolitan area.

However, this study has limitations, including the restriction of sampling to specific provinces, limited clinical follow-up, and sequencing of only a subset of positive samples, which may not capture the full genetic diversity of circulating FeCoVs. The lack of recombination analysis (e.g., SimPlot, RDP4) also limits conclusions about historical recombination events.

Future studies should expand sampling to additional regions of Thailand, incorporate longitudinal monitoring to track genotype shifts, and perform detailed recombination analyses—particularly focusing on the S gene and ORF1ab regions. Cross-species investigations involving canine coronavirus (CCoV) would also help elucidate potential recombination pathways and interspecies transmission risks.

In conclusion, this study establishes essential baseline genomic evidence for FeCoV in Thailand, enhances understanding of circulating biotypes and their evolutionary context, and provides foundational data to improve diagnostic tools, support vaccine development, and inform long-term surveillance strategies. These findings contribute significantly to regional and global efforts aimed at controlling FeCoV infections and mitigating their impact on feline health.

## DATA AVAILABILITY

The nucleotide sequence data are available in the GenBank database under accession numbers # PV797400–409. The supplementary data can be available from the corresponding author upon request.

## AUTHORS’ CONTRIBUTIONS

YNT: Drafted and revised the manuscript. YNT, KC, CN, and EC: Collected the samples. YNT, WJ, EMP, HWP, and SC: Performed virus detection, whole-genome characterization, and phylogenetic analysis. YNT, KC, CN, EC, SP, and SC: Participated in the phylogenetic and genetic analyses. AA: Designed the study, performed data analysis, and drafted, and revised the manuscript. All authors have read and approved the final version of the manuscript.
